# Supplementary material for: Multi‐dataset identification of innovative feature genes and molecular mechanisms in keratoconus
Source: J Cell Mol Med. 2024 Sep 19;28(18):e70079. doi: 10.1111/jcmm.70079 (PMC11412914; doi:10.1111/jcmm.70079)
Supplement: Supplementary file 2 — Material S2. [file JCMM-28-e70079-s002.zip › 7.GSEA analysis/2.ATOH7/KEGG_ADIPOCYTOKINE_SIGNALING_PATHWAY.html]

Details for gene set KEGG\_ADIPOCYTOKINE\_SIGNALING\_PATHWAY[GSEA]

|  || Dataset | ATOH7.group.cls#h\_versus\_l |
| Phenotype | group.cls#h\_versus\_l |
| Upregulated in class | h |
| GeneSet | KEGG\_ADIPOCYTOKINE\_SIGNALING\_PATHWAY |
| Enrichment Score (ES) | 0.48974562 |
| Normalized Enrichment Score (NES) | 1.7280163 |
| Nominal p-value | 0.0060362173 |
| FDR q-value | 0.34396347 |
| FWER p-Value | 0.32 |
Table: GSEA Results Summary

  

Fig 1: Enrichment plot: KEGG\_ADIPOCYTOKINE\_SIGNALING\_PATHWAY      
 Profile of the Running ES Score & Positions of GeneSet Members on the Rank Ordered List

  

| SYMBOL | TITLE | RANK IN GENE LIST | RANK METRIC SCORE | RUNNING ES | CORE ENRICHMENT || 1 | AKT3 | na | 9 | 1.043 | 0.1163 | Yes |
| 2 | IRS1 | na | 55 | 0.848 | 0.2098 | Yes |
| 3 | NPY | na | 136 | 0.737 | 0.2898 | Yes |
| 4 | POMC | na | 897 | 0.464 | 0.3198 | Yes |
| 5 | RXRG | na | 913 | 0.462 | 0.3709 | Yes |
| 6 | PRKAA2 | na | 2242 | 0.326 | 0.3691 | Yes |
| 7 | PCK2 | na | 2919 | 0.285 | 0.3814 | Yes |
| 8 | IRS2 | na | 3662 | 0.249 | 0.3878 | Yes |
| 9 | SLC2A4 | na | 4197 | 0.229 | 0.3980 | Yes |
| 10 | TNFRSF1B | na | 4331 | 0.224 | 0.4192 | Yes |
| 11 | NFKBIE | na | 5212 | 0.194 | 0.4155 | Yes |
| 12 | SOCS3 | na | 5926 | 0.173 | 0.4143 | Yes |
| 13 | CD36 | na | 6081 | 0.169 | 0.4287 | Yes |
| 14 | RELA | na | 6248 | 0.164 | 0.4422 | Yes |
| 15 | LEPR | na | 6380 | 0.160 | 0.4563 | Yes |
| 16 | PPARGC1A | na | 6498 | 0.157 | 0.4705 | Yes |
| 17 | PCK1 | na | 6929 | 0.145 | 0.4742 | Yes |
| 18 | SLC2A1 | na | 6979 | 0.144 | 0.4888 | Yes |
| 19 | ACSL6 | na | 7613 | 0.128 | 0.4849 | Yes |
| 20 | ACSL4 | na | 8350 | 0.111 | 0.4761 | Yes |
| 21 | NFKB1 | na | 8680 | 0.105 | 0.4783 | Yes |
| 22 | G6PC2 | na | 8689 | 0.105 | 0.4897 | Yes |
| 23 | ACSL5 | na | 9868 | 0.083 | 0.4651 | No |
| 24 | JAK2 | na | 10386 | 0.075 | 0.4586 | No |
| 25 | ACSL3 | na | 10431 | 0.075 | 0.4657 | No |
| 26 | NFKBIA | na | 10503 | 0.073 | 0.4718 | No |
| 27 | STAT3 | na | 11584 | 0.058 | 0.4471 | No |
| 28 | PRKAB2 | na | 11761 | 0.056 | 0.4483 | No |
| 29 | TNFRSF1A | na | 11768 | 0.055 | 0.4543 | No |
| 30 | ADIPOR2 | na | 11835 | 0.055 | 0.4585 | No |
| 31 | RXRB | na | 12340 | 0.048 | 0.4493 | No |
| 32 | PRKAB1 | na | 12927 | 0.041 | 0.4370 | No |
| 33 | NFKBIB | na | 13350 | 0.037 | 0.4290 | No |
| 34 | ADIPOQ | na | 13485 | 0.035 | 0.4291 | No |
| 35 | CAMKK2 | na | 14282 | 0.028 | 0.4092 | No |
| 36 | TNF | na | 14399 | 0.027 | 0.4088 | No |
| 37 | IKBKG | na | 15619 | 0.016 | 0.3754 | No |
| 38 | AKT1 | na | 15787 | 0.014 | 0.3722 | No |
| 39 | PRKAG1 | na | 16803 | 0.006 | 0.3436 | No |
| 40 | MAPK8 | na | 17140 | 0.003 | 0.3343 | No |
| 41 | PRKAG3 | na | 17215 | 0.003 | 0.3325 | No |
| 42 | ACACB | na | 17889 | -0.003 | 0.3134 | No |
| 43 | PRKAA1 | na | 18507 | -0.009 | 0.2965 | No |
| 44 | ADIPOR1 | na | 18515 | -0.009 | 0.2973 | No |
| 45 | TRAF2 | na | 19649 | -0.018 | 0.2667 | No |
| 46 | TRADD | na | 19975 | -0.022 | 0.2597 | No |
| 47 | AGRP | na | 20146 | -0.023 | 0.2574 | No |
| 48 | CHUK | na | 20245 | -0.024 | 0.2573 | No |
| 49 | CPT1C | na | 20712 | -0.029 | 0.2470 | No |
| 50 | MAPK10 | na | 20989 | -0.032 | 0.2426 | No |
| 51 | RXRA | na | 21798 | -0.041 | 0.2238 | No |
| 52 | PRKAG2 | na | 22217 | -0.045 | 0.2168 | No |
| 53 | PTPN11 | na | 22613 | -0.050 | 0.2110 | No |
| 54 | IRS4 | na | 23255 | -0.058 | 0.1990 | No |
| 55 | IKBKB | na | 23356 | -0.060 | 0.2028 | No |
| 56 | ACSL1 | na | 23435 | -0.061 | 0.2074 | No |
| 57 | PRKCQ | na | 23761 | -0.066 | 0.2054 | No |
| 58 | CPT1A | na | 23792 | -0.067 | 0.2120 | No |
| 59 | STK11 | na | 23820 | -0.067 | 0.2187 | No |
| 60 | CPT1B | na | 24648 | -0.080 | 0.2037 | No |
| 61 | PPARA | na | 24700 | -0.081 | 0.2113 | No |
| 62 | MAPK9 | na | 24826 | -0.082 | 0.2169 | No |
| 63 | MTOR | na | 25211 | -0.089 | 0.2157 | No |
| 64 | AKT2 | na | 27111 | -0.128 | 0.1753 | No |
| 65 | CAMKK1 | na | 28807 | -0.173 | 0.1457 | No |
| 66 | LEP | na | 30372 | -0.224 | 0.1257 | No |
Table: GSEA details [plain text format]

  

Fig 2: KEGG\_ADIPOCYTOKINE\_SIGNALING\_PATHWAY      
 Blue-Pink O' Gram in the Space of the Analyzed GeneSet

  

Fig 3: KEGG\_ADIPOCYTOKINE\_SIGNALING\_PATHWAY: Random ES distribution      
 Gene set null distribution of ES for **KEGG\_ADIPOCYTOKINE\_SIGNALING\_PATHWAY**
